# Impacts of current and future large dams on the geographic range connectivity of freshwater fish worldwide

**DOI:** 10.1073/pnas.1912776117

**Published:** 2020-02-03

**Authors:** Valerio Barbarossa, Rafael J. P. Schmitt, Mark A. J. Huijbregts, Christiane Zarfl, Henry King, Aafke M. Schipper

**Affiliations:** ^a^Department of Environmental Science, Institute for Water and Wetland Research, Radboud University, 6500 GL Nijmegen, The Netherlands;; ^b^Department of Nature and Rural Areas, PBL Netherlands Environmental Assessment Agency, 2500 GH The Hague, The Netherlands;; ^c^Natural Capital Project, Stanford University, Stanford, CA 94305;; ^d^The Woods Institute for the Environment, Stanford University, Stanford, CA 94305;; ^e^Center for Applied Geoscience, Eberhard Karls University of Tübingen, 72074 Tübingen, Germany;; ^f^Safety and Environmental Assurance Centre, Unilever R&D, Unilever, Sharnbrook MK44 1LQ, United Kingdom

**Keywords:** habitat fragmentation, hydropower, river management, migratory fish, biodiversity

## Abstract

Dams contribute to water security, energy supply, and flood protection but also fragment habitats of freshwater species. Yet, a global species-level assessment of dam-induced fragmentation is lacking. Here, we assessed the degree of fragmentation of the occurrence ranges of ∼10,000 lotic fish species worldwide due to ∼40,000 existing large dams and ∼3,700 additional future large hydropower dams. Per river basin, we quantified a connectivity index (CI) for each fish species by combining its occurrence range with a high-resolution hydrography and the locations of the dams. Ranges of nondiadromous fish species were more fragmented (less connected) (CI = 73 ± 28%; mean ± SD) than ranges of diadromous species (CI = 86 ± 19%). Current levels of fragmentation were highest in the United States, Europe, South Africa, India, and China. Increases in fragmentation due to future dams were especially high in the tropics, with declines in CI of ∼20 to 40 percentage points on average across the species in the Amazon, Niger, Congo, Salween, and Mekong basins. Our assessment can guide river management at multiple scales and in various domains, including strategic hydropower planning, identification of species and basins at risk, and prioritization of restoration measures, such as dam removal and construction of fish bypasses.

## Significance

Freshwater fish are highly threatened by dams that disrupt the longitudinal connectivity of rivers and may consequently impede fish movements to feeding and spawning grounds. In a comprehensive global analysis covering ∼10,000 freshwater fish species and ∼40,000 existing large dams we identified the most disconnected geographical ranges for species in the United States, Europe, South Africa, India, and China. The completion of near-future plans for ∼3,700 large hydropower dams will greatly increase habitat fragmentation in (sub)tropical river basins, where many livelihoods depend on inland fisheries. Our assessment can support infrastructure planning on multiple scales and assist in setting conservation priorities for species and basins at risk.

Freshwater habitats cover only about 0.8% of Earth’s surface, yet they host a disproportionately high diversity of species. One-third of the described vertebrates, including ∼40% of the fish species, are found in freshwater environments (1). Freshwater biodiversity is also disproportionately threatened, with decline rates higher than observed for marine or terrestrial biodiversity (2). Damming of rivers is one of the main threats to freshwater biodiversity (3, 4). While dams provide direct economic benefits (e.g., by contributing to water security, flood protection, and renewable energy), they affect freshwater ecosystems by inundation, hydrologic alteration, and fragmentation, for example (5, 6). Fragmentation of the freshwater environment has major implications for freshwater fish as dams obstruct migration routes, essential for spawning or feeding, and limit dispersal (7, 8, 9). The near-future expansion of hydropower facilities will further threaten freshwater fish biodiversity (4). While an estimated ∼50% of the river volume is currently altered by either flow regulation or fragmentation, the pending construction of ∼3,700 major hydropower dams is expected to increase this percentage to 93% (10, 11).

Large-scale, species-level assessments of current and future freshwater habitat fragmentation are key to highlight remaining and endangered hotspots of biodiversity and to identify and prioritize conservation needs. So far, however, efforts to quantify dam impacts on habitat connectivity have been mainly carried out at local scales (e.g., refs. 9, 12, and 13). Existing global assessments have focused on mapping river connectivity, but without quantifying impacts on freshwater biodiversity (5, 10, 14). An exception is the study conducted by Liermann et al. (15), which related the degree of present-day river fragmentation within freshwater ecoregions to their overall freshwater fish diversity. However, freshwater ecoregions cover large extents (average area = 311,605 km^2^, *n* = 426) (16) and do not account for the actual geographical ranges of species, which can be limited to smaller areas and hence be subject to different degrees of fragmentation. In addition, by encompassing and cutting through multiple watersheds (16), ecoregions do not account for the spatial connectivity of rivers, which defines the spatial template for aquatic biodiversity and species migration. Thus, a spatially resolved and species-specific assessment of fragmentation effects, accounting for the actual global drainage network, is missing.

Here, we assessed impacts of current and future large dams on the geographic range connectivity of ∼10,000 lotic (i.e., living partially or exclusively in flowing freshwater bodies) fish species worldwide. We employed a species-specific modeling approach to quantify connectivity for each fish species based on its geographic occurrence range. We based our analysis on a high-resolution hydrological network comprising ∼1 million subbasin units with an average size of ∼100 km^2^ (17). Based on global dam datasets currently available, we considered ∼40,000 existing large dams (18, 19) and ∼3,700 large hydropower dams (>1 MW) that are currently under construction or planned (11). To estimate the additional impacts of small dams, we employed more detailed regional datasets as available for Brazil, the greater Mekong area, and the United States. We adopted connectivity measures specific to nondiadromous and diadromous fish species, respectively, as fragmentation impacts of dams might differ between fish that migrate between freshwater and marine environments and fish that complete their lifecycle in freshwater (20). The species-based modeling approach combined with a detailed hydrography allowed us to identify species and species groups most at risk as well as geographic hotspots of current and future fragmentation.

## Results

**Fig. 1.**
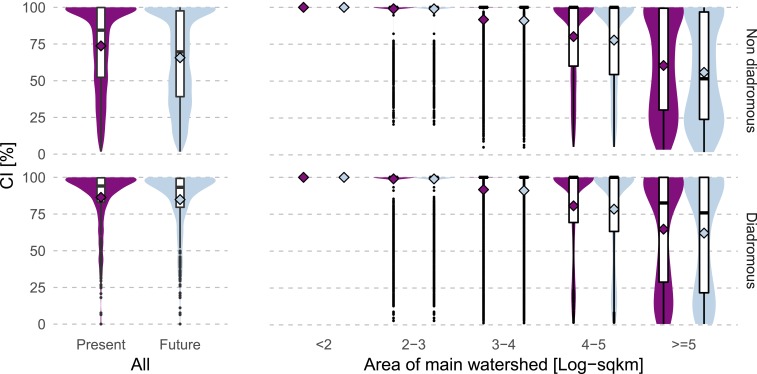
Connectivity index (CI) across species (*Left*) and main hydrologic basins of different size (*Right*) for nondiadromous (*Top*) and diadromous (*Bottom*) fish species. Values are shown for present dams (purple) as well as present and future dams together (light blue). Main hydrologic basins are defined as having an outlet to the sea or internal sink. For species occurring in multiple main basins, the area-weighted mean of the basin-specific CI values was calculated (*Top*). The basin-level CI (*Right*) represents the mean of the CI values across the species occurring within the basin. Boxes represent the interquartile range and the median, and whiskers the 95% interval. Colored violins around the boxes show the values distribution. Diamonds represent the mean.

**Fig. 2.**
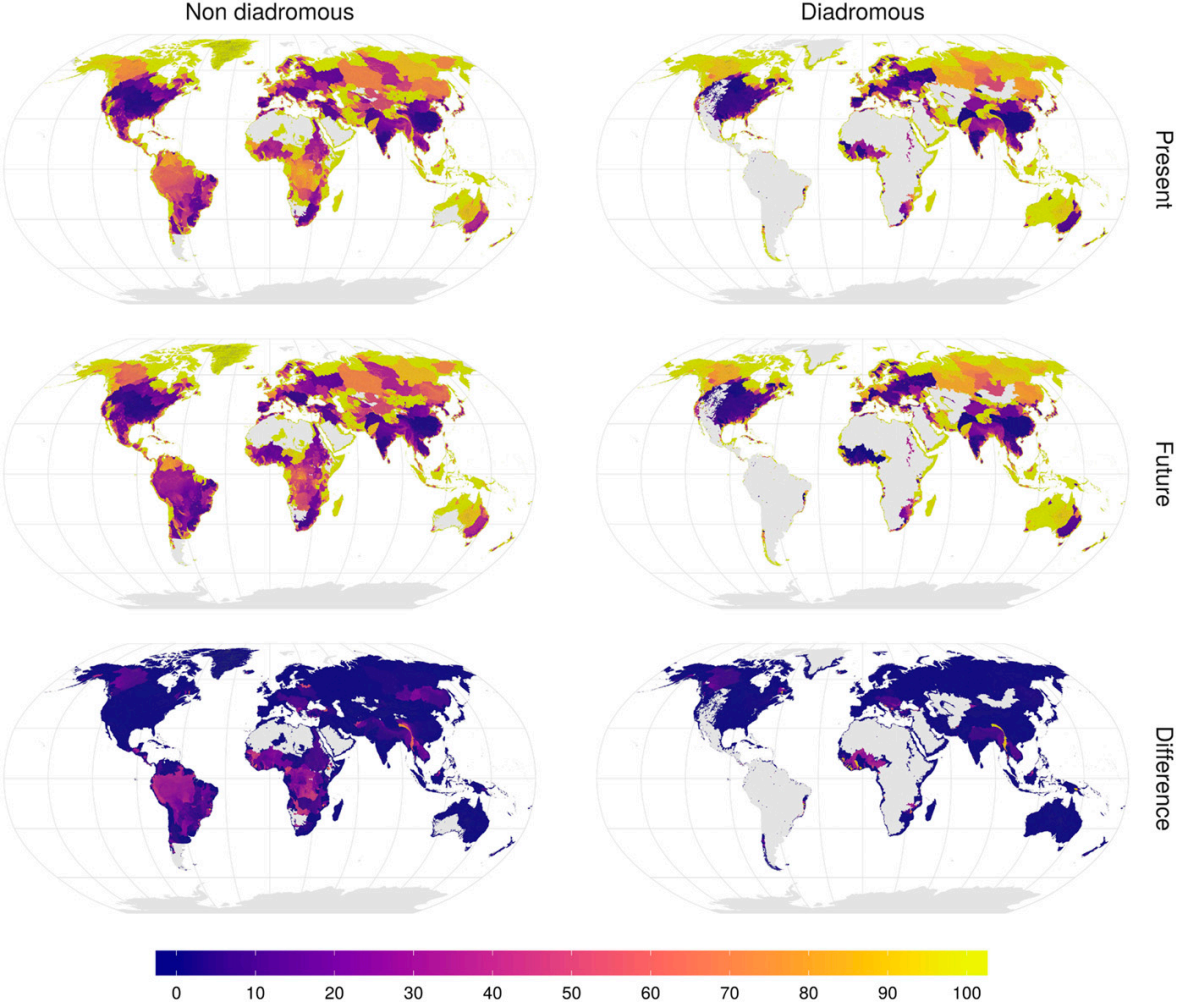
Mean Connectivity Index (CI in percent) per subbasin (∼1 million units) for present situation (*Top*), future projection (*Center*), and the difference between them (*Bottom*) for nondiadromous (*Left*) and diadromous (*Right*) fish species. Gray represents areas without species range data.

Based on the ∼40,000 existing large dams, we found average range connectivity values of 73 ± 28% for nondiadromous and 86 ± 19% for diadromous species (mean connectivity index [CI] ± SD) (Fig. 1). The completion of ∼3,700 hydropower dams that are currently under construction or planned further reduces the connectivity of nondiadromous species’ ranges (mean CI = 66 ± 30%; Fig. 1). For diadromous species the average future decrease in CI was smaller (mean CI = 85 ± 21%; Fig. 1) but still locally relevant (Fig. 2). Both current and future range connectivity were lower for species in larger than in smaller river basins (Fig. 1).

**Fig. 3.**
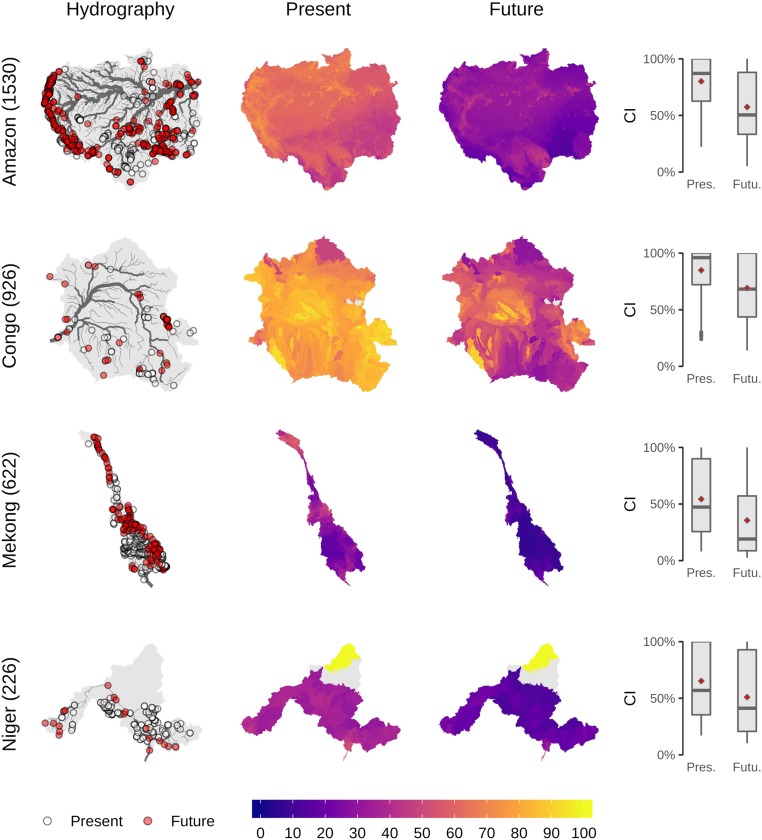
Mean Connectivity Index (CI in percent) across nondiadromous species in four exemplary main hydrologic basins. The maps show the basin hydrography with the locations of dams (*Left*) and the CI at the subbasin level for the present situation and future projection (*Center*). The species-specific CI values are summarized as boxplots, with diamonds representing the mean (*Right*). Numbers in brackets next to each basin name represent the number of fish species considered. Locations of the selected basins are shown in *SI Appendix*, Fig. S7.

Our results revealed the lowest CI values for species occurring in the United States, Europe, South Africa, India, and China. These values did not substantially decrease in the future (Fig. 2). We found the largest differences between impacts of present and future dams for species occurring in South America, Africa, and Southeast Asia (Fig. 2). Decreases in connectivity due to future dams were particularly large for nondiadromous species in large tropical and subtropical rivers, for example the Amazon, Congo, Niger, Salween, and Mekong (Figs. 2 and 3*A* and *SI Appendix*, Fig. S6). For instance, we found that the mean CI across the nondiadromous species of the Amazon basin dropped by ∼30 percentage points in the future, by ∼20 percentage points in the Mekong, Congo, and Niger and by ∼40 percentage points in the Salween (Fig. 3).

**Fig. 4.**
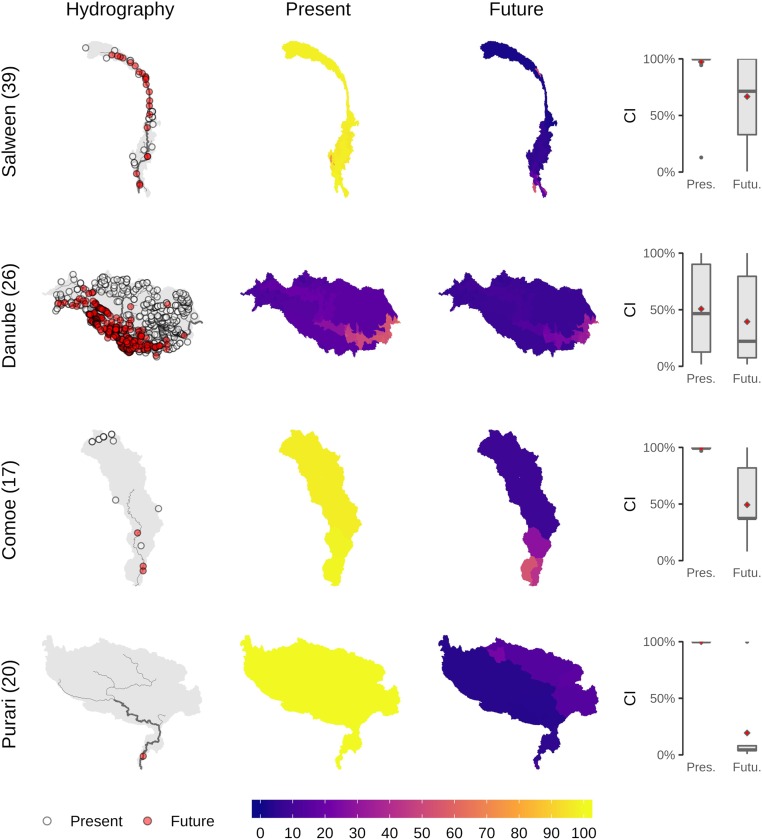
Mean connectivity index (CI in percent) across diadromous species for four exemplary main hydrologic basins. The maps show the basin hydrography with the locations of dams (*Left*) and the CI at the subbasin level for the present situation and future projection (*Center*). The species-specific CI values are summarized as boxplots, with diamonds representing the mean (*Right*). Numbers in brackets next to each basin name represent the number of fish species considered. Locations of the selected basins are shown in *SI Appendix*, Fig. S7.

Our results indicated that the range connectivity of diadromous species declined most due to future dams in small basins along the coastline of central-eastern Africa, western Africa, and the Malay Archipelago (Fig. 2). For instance, in the Comoe in western Africa and the Purari basin in Papua New Guinea, the construction of a few mainstream dams resulted in a strong decrease in connectivity (Fig. 4). The mean connectivity dropped from ∼100 to ∼20% in the Purari basin and from 100% to ∼50% in the Comoe basin (Fig. 4). Yet, large reductions in CI for diadromous species were not limited to small basins but were also observed in larger basins like the Danube, Niger, and Salween, where connectivity dropped as much as ∼40 percentage points (Figs. 2 and 4 and *SI Appendix*, Fig. S6).

**Fig. 5.**
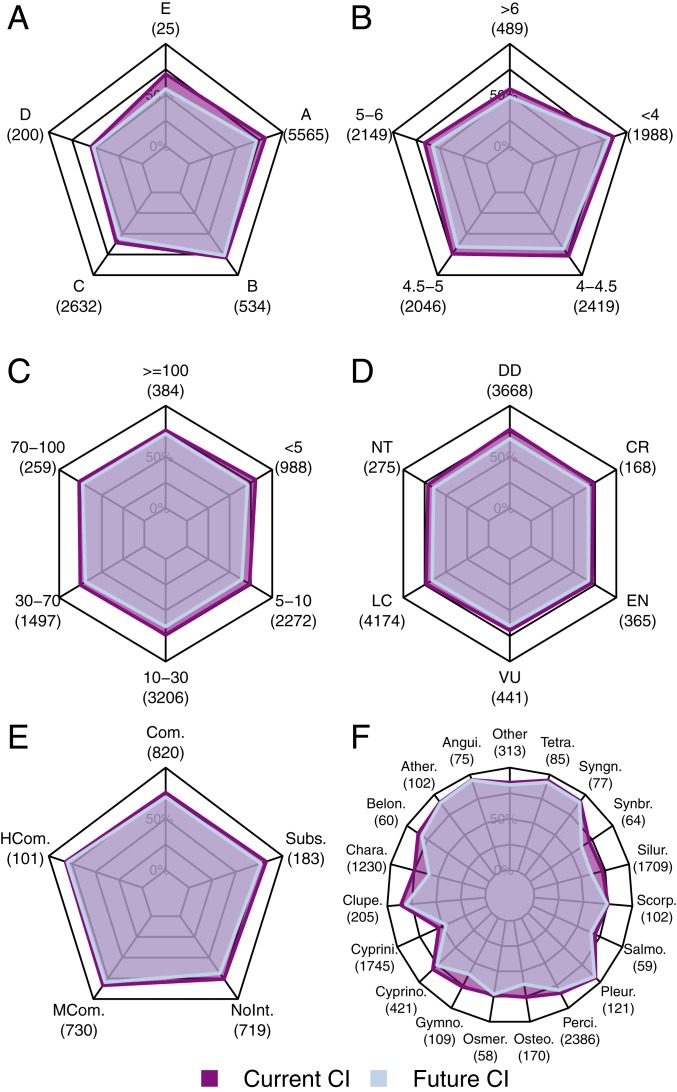
Mean of species-specific connectivity index (CI in percent) values by different traits and categories for present and future, including (*A*) Köppen–Geiger climate zones, where A = equatorial, B = arid, C = warm temperate, D = snow, and E = polar. (*B*) Geographic range area (log [base 10]-transformed square kilometers). (*C*) Body length (centimeters). (*D*) IUCN threat status, where CR = critically endangered, VU = vulnerable, NT = near threatened, LC = least concern, EN = endangered, and DD = data deficient. (*E*) Commercial relevance, where Com. = commercial, HCom. = highly commercial, MCom. = minor commercial, NonInt. = of no interest, and Subs. = subsistence fisheries. (*F*) Species order, where groups with less than 20 species are grouped together in the “other” category (the full list of order names is provided in *SI Appendix*, Table S2). For species occurring in multiple main hydrologic basins, an area-weighted mean of the basin-specific CI values was calculated before averaging across species. Each panel shows a subset of the analyzed 9,794 species in this study (underneath each category the number of species is reported in brackets), as metadata for species traits and categories were not available for all of the species.

Currently, the occurrence ranges of species in tropical rivers (*n* = 5,565) were the most connected, but the connectivity dropped considerably in response to the construction of future dams (mean CI from 81 to 71%; Fig. 5*A*). In contrast, ranges of the 2,632 species found in temperate climates were the most fragmented by current dams, but the decline in connectivity due to future dams was lower (mean CI from 60 to 55%; Fig. 5*A*). Larger occurrence ranges (>∼10,000 km^2^) were characterized by lower connectivity values, which could further decrease in the future compared to smaller ranges (Fig. 5*B*). We found the least range fragmentation for very small species (body length <5 cm; mean CI = 80%) and slightly more for the remaining species (body length >5 cm; mean CI between 74% and 77%; Fig. 5*C*). Species classified as “least concern” or “not threatened” by the International Union for Conservation of Nature (IUCN) were characterized by CI values equal to or lower than those of threatened species and slightly higher decreases due to future dams (Fig. 5*D*). Furthermore, we found slightly higher mean CI values for species of commercial importance (∼76 to 83%) compared to species of no commercial interest (mean CI = 76%), while projected future declines in connectivity were relatively large for species of no commercial interest and species subject to subsistence fisheries (decline in mean CI of 6 to 7 percentage points; Fig. 5*E*). Ranges of species belonging to the Cypriniformes order stood out in terms of habitat fragmentation, with a mean CI of 54% and a projected decrease due to future dams of 8 percentage points. Other taxonomic groups with already low CI values included Salmoniformes (mean CI = 67%) and Synbranchiformes, Siluriformes, Osmeriformes, Characiformes, Scorpaeniformes, Osteoglossiformes, and Gymnotiformes (mean CI = 72 to 75%) (Fig. 5*F*). For most of these same taxonomic groups, we also found the largest declines in range connectivity due to future dams’ construction.

**Fig. 6.**
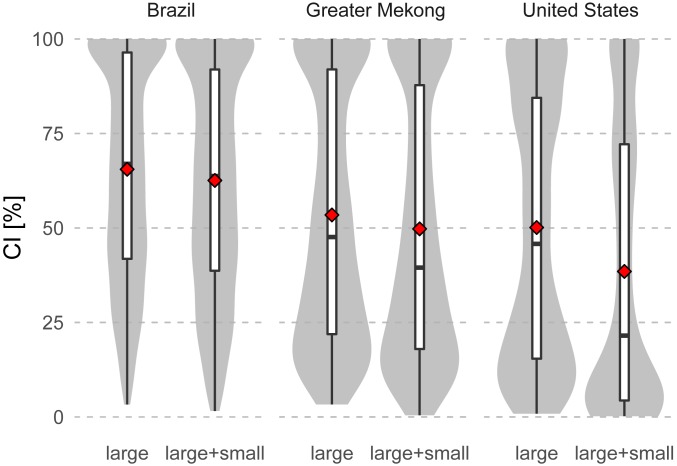
Effect of small dams on the CI values across freshwater fish species in Brazil, the greater Mekong region, and the United States. The comparison is made for each region by considering only large dams (consisting of dams employed in this study complemented with large dams [height >15 m] from national datasets; left) and by adding small dams from national datasets (right). Boxes represent the interquartile range and the median and whiskers the 95% interval. Gray areas around the boxes show the values distribution. The red diamonds represent the mean.

Our global analysis focused on large dams, reflecting a lack of global datasets on existing and future small barriers. To provide insight into the combined impacts of large and small dams, we quantified the potential additional effect of small dams in Brazil, the greater Mekong region (combined Mekong–Irrawaddy–Salween basins), and the United States, based on national and regional datasets (Fig. 6). In this comparison, we included 1,996 small hydropower dams for Brazil, 544 small dams for the greater Mekong, and 64,449 small dams for the United States (see *SI Appendix* for further information on the dams’ datasets). The addition of small dams resulted in additional declines in mean CI of 3, 4, and 12 percentage points for Brazil, the greater Mekong region, and the United States, respectively (Fig. 6).

## Discussion

In this study we assessed impacts of present and future dams on the connectivity of the occurrence ranges of ∼10,000 lotic fish species. Based on the largest consistent global compilation of existing large dams, we found that the ranges of nondiadromous species are, on average, considerably more fragmented than the ranges of diadromous species (Fig. 1). The marked difference is likely due to the different spatial distribution of the two species groups with respect to the locations of existing and future dams (*SI Appendix*, Figs. S4 and S5). While ranges of diadromous species are highly fragmented in Europe and North America, hotspots of diadromous species richness occur in small basins of the Malay Archipelago and African coastline (*SI Appendix*, Fig. S4), which are less fragmented (Fig. 2 and *SI Appendix*, Fig. S5). The connectivity reduction of ∼7 percentage points for nondiadromous species (Fig. 1), instead, suggests that the relatively large number of upstream dams planned for hydropower generation (*SI Appendix*, Fig. S9) (11) will affect nondiadromous fish disproportionately. According to our results, ranges of many nondiadromous species in the world’s largest tropical basins will become highly fragmented after the completion of dams that are currently under construction or planned (Figs. 2 and 3). This is in line with the general expectation that the biodiverse tropical basins such as the Amazon, Mekong, and Congo will experience large ecological consequences from the expected boom in hydropower in these regions (21, 22).

Our global analysis focused on large dams, as only these data are consistently assembled and available on a global scale (5, 18, 19). However, small dams cumulatively have a considerable additional impact on the connectivity of freshwater fish species’ ranges (ref. 23 and Fig. 6). Hence, our global connectivity assessment represents a best-case estimate. The magnitude of the additional impacts of small dams is likely to differ among regions and basins, as exemplified by our results for Brazil, the greater Mekong, and the United States. For the United States, with a long legacy of hydraulic engineering, small dams have a major impact on range connectivity. In the greater Mekong and Brazil, impacts of small dams are still rather small. Our regional results highlight that locating and georeferencing existing and planned small dams is an important future task for planning freshwater conservation. This holds particularly for regions with a possible massive expansion in small hydropower, such as Russia, China, or South America (23), as well as small basins that are still highly connected and, while there are no plans for large dams, plans for local development of small infrastructure are mostly unknown.

Our results may further underestimate fragmentation impacts because present-day species ranges may have already been contracted compared to a pristine situation due to existing dams (9). The only factor that could lead to an overestimation of fragmentation is that we assumed barriers fully impassable, whereas fragmentation could be mitigated by fish passes. Yet, while efforts to include fish passes are progressing, evidence suggests that fish passes are selective for specific species and sometimes even harmful, with nature-like bypasses as the only, but rarely implemented, exception (24, 25). We further acknowledge that our approach is based on subbasin units rather than on the actual river network, reflecting the geographical range data for freshwater fish as provided by the IUCN. While we approximate the corresponding river length following Hack’s law, future improvements may include using actual estimates of total river length per subbasin. Yet, challenges remain in mapping headwaters (i.e., the most upstream termination of the stream) as well as intermittent river segments. These challenges need to be addressed to correctly estimate the actual river network, and in turn total river length, in digital elevation model-derived products such as HydroSHEDS (17, 26, 27).

Longitudinal fragmentation has been identified as a leading cause of freshwater fish habitat degradation (3, 4, 7). Yet, our results revealed no clear difference in range connectivity between species of different IUCN threat categories (Fig. 5*D*). This might reflect a confounding effect of range size, as we found larger fragmentation for species with larger geographical ranges, whereas IUCN extinction risk decreases with range size (28). Moreover, additional stressors, such as water pollution, flow alteration, and overfishing, may also influence species’ extinction risks (4). Increased levels of fragmentation could potentially exacerbate the ecological effects of size-biased harvesting of freshwater fish species. Medium to large species have, on average, a slightly more fragmented range than very small species, meaning that existing dams could have increased the impacts of direct human exploitation, which are larger on the bigger fish species (29). Yet, future hydropower dams will disproportionally increase the degree of range fragmentation of medium to small species, which account for more than a half of the overall freshwater fish diversity. These results highlight the need for expanding research efforts to small and medium-sized species, which might be at higher risk of habitat degradation due to future dam-driven longitudinal fragmentation.

Higher levels of fragmentation will likely reduce fish populations (30–32). By disconnecting the continuum of the river network, dams isolate populations, reduce access to feeding areas, and disrupt access to spawning sites (8, 9). Indirectly, dams also exert additional upstream and downstream pressures on the aquatic habitats and species, for example by altering flow and thermal regime as well as sediment and nutrient dynamics (5, 6, 33). For example, changes in flow regime may affect diadromous fish species by altering the conditions required for the transitions to and from the ocean (34, 35). Moreover, dams may also affect the lateral connectivity between floodplains and rivers, which in turn may influence floodplain ecosystem dynamics and productivity (36, 37). Thus, dams create trade-offs between the provision of energy and water management services and impacts on ecosystems, which might eventually result in high socioeconomic costs (38). This holds especially where communities are highly reliant on inland fisheries as source of proteins and household income, for example in basins such as the Amazon, Congo, Niger, Mekong, Irrawaddy, and Salween (38–40). Aquaculture in reservoirs might possibly compensate some reduction in fish catch but comes with additional environmental externalities (41, 42).

Our species-specific and high-resolution method (subbasin units of ∼100 km^2^) enables further understanding of potential ecological effects of existing and future dams from catchment to regional and global scales. This information can inform strategic planning of future dams or prioritization of conservation measures. For instance, the case of the Purari basin in Papua New Guinea showed that the potential completion of one downstream dam could strongly reduce the connectivity of the basin for diadromous species (Fig. 4). By only considering the topologic connectivity of the river network, without accounting for the actual geographical ranges and migratory behavior of species, such patterns would not emerge. Our assessment also showed that fish species in western Europe, the United States, India, and China retain the lowest connectivity values (Fig. 2). In Europe and the United States, efforts to remove ecologically impactful dams and restore longitudinal connectivity are currently underway. For instance, ∼1,500, mostly small, dams have already been removed in the United States (43, 44) and ∼2,500 in Europe (data from the United Kingdom, Sweden, Spain, and Finland; https://www.damremoval.eu/), although potential ecological trade-offs related to the removal of large dams are still not well understood (45, 46). Our study can aid designing optimization strategies to prioritize sites for hydropower expansion (47) as well as river restoration, for example through dam removal (48) or nature-like bypass construction (25), to minimize or reduce ecological impacts and maximize benefits of dams.

## Methods

### Geographic Ranges of Fish Species.

Our analysis focuses on lotic species (i.e., that are found in flowing water bodies) and we thus excluded lentic species that occur exclusively in stagnant water bodies. We compiled geographical ranges for 9,794 lotic freshwater fish species from two sources (*SI Appendix*, Fig. S4). The IUCN Red List of Threatened Species (49) provides geographical ranges for 7,242 freshwater fish species, about half of the known freshwater fish species (50), compiled from occurrence records and expert knowledge. Among these, we selected 5,638 lotic species reported as extant. We complemented the IUCN ranges by compiling geographical ranges using point occurrence records from multiple sources, resulting in 4,156 additional freshwater lotic species ranges (see *SI Appendix* for details on the occurrence records datasets). To compile the ranges we followed the same procedure as the IUCN, that is, we merged HydroBASINS units/subbasins (51) containing one or more point occurrence records of the species (see *SI Appendix* for more details on the procedure). We included only species with at least 10 point occurrence records available in the complementary dataset, in line with Warren et al. (52, 53). Our final dataset included 9,794 species, which we classified based on migratory behavior using information from FishBase (https://www.fishbase.in/search.php) (54). We classified species that migrate to/from the marine environment as “diadromous” (*n* = 490) and the remaining as “nondiadromous” (*n* = 9,304).

### Present and Future Dam Locations.

We retrieved data on the locations of 39,912 large dams worldwide (*SI Appendix*, Fig. S5), including 7,320 dams from the Global Reservoir and Dam (GRanD) database (18) and 32,592 additional dams from the GlObal geOreferenced Database of Dams (GOODD), comprising dams visible on global remote-sensing imagery (19). To date, these two databases represent the most comprehensive global source for georeferenced data on large dams (5). We used the Future Hydropower Reservoirs and Dams (FHReD) database for future dam locations (11), which includes 3,681 dams (from here on called “future dams”) of which 574 are “under construction” while the others are planned. This collection of dams is limited to hydropower dams above 1-MW capacity with available data on location and capacity and which have, at least, passed the feasibility evaluation stage (11). In addition to the global GRanD and GOODD datasets, we also retrieved data on large and small dams from national and regional datasets. These included 70,182 dams for the United States, 773 dams for the greater Mekong area, and 2,494 hydropower dams for Brazil (details on data sources and cleaning are provided in *SI Appendix*). We note that we do not account for natural barriers or discontinuities (i.e., waterfalls) in our analysis, reasoning that as waterfalls evolve over evolutionary timescales species will either adapt to the discontinuity or be subject to allopatric speciation, leading to having different species on the two sides of the waterfall (55). Hence, waterfalls are not expected to cause range fragmentation.

### CI Calculation.

We referenced the geographical ranges of each species to any overlapping subbasin unit from the ∼1 million HydroBASINS subbasin units (Pfafstetter level 12, median area = 137 km^2^), hereafter simply called subbasins, which represent the hydrological unit used to map the connectivity (51). Both IUCN and the complementary geographical ranges developed in this study consist of polygons mapped based on the same HydroBASINS subbasin units at a coarser level of aggregation (Pfafstetter level 8), thereby ensuring a perfect overlap with the smaller subbasins employed in this study. Thus, we determined in which subbasins a species occurs and, in turn, all species occurring in any given subbasin.

We calculated a range CI for each species following the approach of Cote et al. (20), differentiating between diadromous and nondiadromous species. In our study, we applied the approach developed for potamodromous species to the nondiadromous category, which includes both resident migratory (potamodromous) and resident nonmigratory lotic species. Connectivity indices are commonly calculated based on vectorized river networks. However, the IUCN database reports species’ occurrence as geographical ranges, areas covering a portion of a main hydrologic basin (which is defined as a basin with an outlet to the sea/internal sink). To apply the connectivity indices to range areas, we converted subbasin area to river length using a well-proven power law $l=\beta a^{\alpha }$ (56, 57). *β* is dependent on the shape of the subbasin, while *α* is a constant ranging between 0.5 and 0.6 (56). When substituting for *l* in the CI equations from ref. 20, *β* cancels out (*SI Appendix*). Hence, we calculated the CI for each nondiadromous fish species *s* in the main hydrologic basin *b* (*CI*^*N*^_*s,b*_) as

$$CI_{s,b}^{N}\;=\;\frac{\sum_{i=1}^{n}{\left(\sum_{j=1}^{m}a_{j,i,s,b}^{\alpha }\right)}^{2}}{{\left(\sum_{i=1}^{n}\sum_{j=1}^{m}a_{j,i,s,b}^{\alpha }\right)}^{2}}\cdot 100,$$ [1]

where *a*_*j,i,s,b*_ represents the area of subbasin *j* belonging to the isolated patch *i* (due to a dam) within a main hydrologic basin *b* and hosting species *s*. We used a value of 0.55 for *α*, that is, the central value within the 0.5 to 0.6 range proposed by ref. 56. The isolated patches are counted from the most downstream to the most upstream patch *n* and are defined as the area upstream a dam or outlet/sink connecting zones of the species geographical range until the next upstream dam or the main basin boundary. The CI of diadromous fish species *s* in basin *b* (*CI*^*D*^_*s,b*_) was calculated as

$$CI_{s,b}^{D}=\frac{\sum_{j=1}^{m}a_{j,1,s,b}^{\alpha }}{\sum_{i=1}^{n}\sum_{j=1}^{m}a_{j,i,s,b}^{\alpha }}\cdot 100.$$ [2]

In this case, the CI for diadromous species is solely dependent on the sum of subbasin areas *a*_*j,1,s,b*_ belonging to the most downstream patch connected to the ocean. This means that the most downstream dam will affect connectivity for diadromous species to a much higher extent than for nondiadromous species (20). The derivation of the equations along with an example sketch as well as additional details on the choice of the underlying hydrography are provided in *SI Appendix*.

### Aggregation of CI Values Across Species.

Based on the species-specific CI values, we calculated a global mean value of CI across all diadromous and nondiadromous species, respectively, for both the present and future scenario. For species occurring in multiple main hydrologic basins (see previous section for a definition of main hydrologic basin), we calculated a mean CI value weighted by the occurrence range area of the species within the different main basins. We further calculated basin-specific CI values as the mean CI across all species occurring in the basin. Finally, we calculated the mean CI for species groups characterized by different traits, again using an area-weighted mean for species occurring in multiple basins. We retrieved data from FishBase (54) on taxonomic group (Order), maximum body length and commercial importance (species of high–low commercial relevance or used in subsistence fisheries). We performed a synonyms check for the binomial nomenclature provided in the IUCN database to maximize the overlap with the FishBase database (54). We differentiated species according to main climate zone by overlaying the occurrence ranges with the Köppen–Geiger climate categories (58). Species falling into multiple climate zones were assigned the climate zone with the largest overlap. We retrieved information on threat status (Red List) from the IUCN application programming interface (49).

### Comparing CI Values among the Different Dam Datasets.

We collected data on large and small dams from national datasets for Brazil, the greater Mekong area (Mekong–Irrawaddy–Salween main hydrologic basins), and the United States (*SI Appendix*, *Supplementary Methods*). We used these additional data to understand the magnitude in CI increases when considering also small barriers. We compared species-specific CI values obtained using either large dams from the GRanD and GOODD complemented with large dams (height >15 m) from the national datasets or all of the dams including small barriers from national datasets. In addition, to check the representativeness of the GRanD and GOODD datasets for large dams, we compared the CI obtained from these global datasets with the CI obtained based on large dams (height >15 m) from the national datasets. We found a good agreement between results for large dams, with differences in average CI of 5, 9, and 3 percentage points for Brazil, the greater Mekong, and the United States, respectively (*SI Appendix*, Fig. S8).

### Data Availability.

The species-specific CI values resulting from our analysis are available as an excel table in Dataset S1. The code used to perform the analyses described by this article and generate the species-specific CI values is freely available at https://github.com/vbarbarossa/connectfish. The code used to generate the additional geographic ranges from point occurrence records is available at https://github.com/vbarbarossa/occ2range4fish.

## Supplementary Material

Supplementary File

Supplementary File
