# Supplementary material for: Impacts of current and future large dams on the geographic range connectivity of freshwater fish worldwide
Source: Proc Natl Acad Sci U S A. 2020 Feb 3;117(7):3648–55. doi: 10.1073/pnas.1912776117 (PMC7035475; doi:10.1073/pnas.1912776117)
Supplement: Supplementary File [file pnas.1912776117.sapp.pdf]

PNAS
www.pnas.org

Supplementary Information for

Impacts of current and future large dams on the geographic range connectivity of freshwater fish worldwide

Valerio Barbarossa[a,b,1], Rafael J. P. Schmitt[c,d], Mark A. J. Huijbregts[a], Christiane Zarfl[e], Henry King[f], Aafke M. Schipper[a,b]

[a]Department of Environmental Science, Institute for Water and Wetland Research, Radboud University, 6500 GL Nijmegen, The Netherlands; [b]Department of Nature and Rural Areas, PBL Netherlands Environmental Assessment Agency, 2500 GH The Hague, The Netherlands; [c]Natural Capital Project, Stanford University, Stanford, CA 94305; [d]The Woods Institute for the Environment, Stanford University, Stanford, CA 94305; [e]Center for Applied Geoscience, Eberhard Karls University of Tübingen, 72074 Tübingen, Germany; and [f]Safety and Environmental Assurance Centre, Unilever R&D, Unilever, Sharnbrook MK44 1LQ, United Kingdom

[1]To whom correspondence may be addressed. Email: v.barbarossa@fnwi.ru.nl.

This PDF file includes:

Supplementary text
Figures S1 to S9
Tables S1 to S2
SI References

www.pnas.org/cgi/doi/10.1073/pnas.1912776117

Supplementary Information Text

Supplementary Methods

Compilation of freshwater fish species occurrence records. We collected point occurrence records from external datasets to complement the IUCN geographical ranges for those species not represented in the IUCN data. We used both global and national datasets. The focus of national datasets was especially centered on enriching species data for South America which is scarcely represented within the IUCN database and yet it represents the most biodiverse hotspot for freshwater fish species. We extracted freshwater fish species from the datasets listed in Table S1 based on freshwater fish species names and associated synonyms provided by fishbase.org, IUCN and Tedesco et al., (1). Table S1 lists the number of species and associated occurrence records extracted from each data source (including synonyms and freshwater fish species with records falling within saltwater areas, i.e., diadromous). When merging the records from the different datasets, we removed duplicates and checked for synonyms by referencing all the species to the names reported by fishbase.org (2). This dataset consisted of 2,427,956 occurrence records for 12,233 freshwater fish species (Figure S1). The code used to extract, clean and merge the species occurrence records is freely accessible at https://github.com/vbarbarossa/occ2range4fish.

Deriving fish ranges from occurrence records. We used the occurrence point records to draw species-specific geographical ranges. We followed the same approach used by IUCN. We first referenced the point occurrence records to the underlying HydroBASINS unit (level 8 of aggregation) (3) and therefore dissolved the corresponding polygons to obtain species-specific geographical ranges. The code used to develop the geographical ranges is available at https://github.com/vbarbarossa/occ2range4fish.

Representability of freshwater fish ranges used in this study. We checked the global coverage of the geographical ranges employed in this study against the most comprehensive list of species by main drainage basin (i.e., with an outlet to the sea or an internal sink) provided by Tedesco et al., (1). To this end, we calculated species richness (i.e., number of unique species) within the main drainage basins as reported in (1). We then calculated a coverage ratio as $SR/SR_{ref}*100$ [%] for each main drainage basin (Figure S2).

Lotic/lentic species classification. We classified species as lotic, lentic or both lotic and lentic, using metadata from the IUCN Red List (4). For each species, we retrieved a list of habitat types where the species was known to be found. We classified species as lotic if they were associated with habitats containing at least one of the categories “river”, “stream”, “creek”, “canal”, “channel”, “delta”, “estuaries”, and as lentic if the habitat descriptions contained at least one of the words “lake”, ”pool”, ”bog”, ”swamp”, ”pond”. For species not present in the IUCN metadata we complemented habitat information from fishbase.org (2). We classified species as lotic and lentic based on the flags “Stream” and “Lakes”, respectively, available for each species from the fishbase.org API (2).

Hydrological units. We employed the HydroBASINS sub-basin units (Pfafstetter level 12) for the underlying hydro-morphology used to calculate the longitudinal connectivity in our analysis (3, 5). Henceforth, we refer to sub-basins as the HydroBASINS units and to the main hydrologic basin as to the connected sub-basins that drain to the sea or an internal sink (Figure S3). We allocated the geographical ranges of each species to the ~1M overlapping sub-basin units so that each sub-basin was assigned a list of species for which it provides habitat. In turn, we identified all sub-basins that provide habitat for each species. HydroBASINS divides the globe in 1,034,083 sub-basins (area median = 135 km^2, interquartile

range = 64 km^2) following the Pfafstetter coding scheme (3) and based on the high-resolution 15 arc-seconds (~500m) HydroSHEDS hydrography (5). We used HydroBASINS as both IUCN and the complementary geographical ranges developed in this study are established based on HydroBASINS sub-basin units at a coarser level of aggregation (Pfafstetter level 8). With the Pfaffstetter level 12 we used the highest level of spatial definition available, i.e., the smallest sub-basin units. Each of the sub-basins carries information on the connectivity to the next downstream sub-basin, which allows to determine the total connected area within a main hydrologic basin. Dams falling within a sub-basin were georeferenced to the downstream boundary of that sub-basin so that isolated patches were a collection of HB sub-basin units (Figure S3).

Derivation of the connectivity index equations. The equations proposed by Cote et al., (2009) allow to calculate the connectivity index for non-diadromous (N) and diadromous (D) fish species, assuming barriers are impassable, as follows:

$$CI_{s,b}^{N} = \frac{\sum_{i=1}^{n} {l_{i,s,b}}^2}{(\sum_{i=1}^{n} l_{i,s,b})^2} \cdot 100 \qquad \text{(Eq. S1)}$$

$$CI_{s,b}^{D} = \frac{l_{1,s,b}}{\sum_{i=1}^{n} l_{i,s,b}} \cdot 100 \qquad \text{(Eq. S2)}$$

where $l_{i,s,b}$ represents the length of stream segment i isolated due to a dam for a species s within a main basin b and n is the number of isolated segments due to $n-1$ dams within that basin. Hence, $CI_{s,b}$ expresses the habitat connectivity, with smaller values indicating less connectivity. The equation for diadromous species differs from the one for non-diadromous as the most downstream dam obstructing the passage to/from the marine environment is likely to have the highest impact (6). In Eq. S2, $l_{1,s,b}$ is the length of the longest river segment that is connected to the ocean. While the measures of Cote et al. (6) can in principle account for different passability of barriers, we assume here that the dams considered in this analysis are impassable.

The species occurrence locations are not reported per stream segment, but as geographical ranges occupying a portion of the hydrologic basin, while Eq. S1-S2 were developed for river segments. To make these equations applicable to the areal range data from IUCN, we propose the following conversion between a sub-basin area and the length of the streams in that area based on Hack's law. According to Hack's law (7), $l = \beta a^{\alpha}$, i.e., the length of a stream (l) is proportional to its drainage area (a). Therefore, Eq. 1 and 2 can be rewritten as:

$$CI_{s,b}^{N} = \frac{\sum_{i=1}^{n}(\sum_{j=1}^{m} \beta {a_{j,i,s,b}}^{\alpha})^2}{(\sum_{i=1}^{n}\sum_{j=1}^{m} \beta {a_{j,i,s,b}}^{\alpha})^2} \cdot 100 \qquad \text{(Eq. S3a)}$$

$$CI_{s,b}^{D} = \frac{\sum_{j=1}^{m} \beta {a_{j,1,s,b}}^{\alpha}}{\sum_{i=1}^{n}\sum_{j=1}^{m} \beta {a_{j,i,s,b}}^{\alpha}} \cdot 100 \qquad \text{(Eq. S4a)}$$

where, *m* are the sub-basin areas from j *to* m within the isolated patch *i* where the species *s* occurs. Eq. S3a and S4a can in turn be rewritten as:

$$CI_{s,b}^{N} = \frac{\beta^2 \sum_{i=1}^{n}(\sum_{j=1}^{m} {a_{j,i,s,b}}^{\alpha})^2}{\beta^2 (\sum_{i=1}^{n}\sum_{j=1}^{m} {a_{j,i,s,b}}^{\alpha})^2} \cdot 100 \qquad \text{(Eq. S3b)}$$

$$CI_{s,b}^{D} = \frac{\beta \sum_{j=1}^{m} {a_{j,1,s,b}}^{\alpha}}{\beta \sum_{i=1}^{n} \sum_{j=1}^{m} {a_{j,i,s,b}}^{\alpha}} \cdot 100 \quad \text{(Eq. S4b)}$$

Therefore, β can be eliminated and the final equations are as follows:

$$CI_{s,b}^{N} = \frac{\sum_{i=1}^{n} (\sum_{j=1}^{m} {a_{j,i,s,b}}^{\alpha})^2}{(\sum_{i=1}^{n} \sum_{j=1}^{m} {a_{j,i,s,b}}^{\alpha})^2} \cdot 100 \quad \text{(Eq. S3c)}$$

$$CI_{s,b}^{D} = \frac{\sum_{j=1}^{m} {a_{j,1,s,b}}^{\alpha}}{\sum_{i=1}^{n} \sum_{j=1}^{m} {a_{j,i,s,b}}^{\alpha}} \cdot 100 \quad \text{(Eq. S4c)}$$

Figure S3 shows an example application of equations S4a and S4c.

National dams' datasets. We retrieved dams from the National Inventory of Dams (NID; https://nid.sec.usace.army.mil/) for the USA, which consists of 91,226 dams above 15 feet or with a major hazard potential for downstream people. Of those, we excluded 21,044 dams used for purposes such as fire protection, stock, small fish ponds, tailings, debris control which are likely off-stream and therefore not directly affecting the longitudinal connectivity of the river network. We split the NID dataset in large (n = 5,733) and small (n = 64,449) dams based on a height threshold of the dam of 15 meters. For the greater Mekong area (Mekong-Irrawaddy-Salween main hydrologic basins), we gathered data on the location of 1,007 dams from https://opendevelopmentmekong.net. We selected 773 dams with latitude-longitude information and that were classified as existing or under construction (Status = "OP", "COMM", "UNCON"). We split the greater Mekong dams in large (n = 229) and small (n = 544) based on the same 15 meters height threshold. For Brazil, we retrieved data on 498 large and additional 1,996 small hydropower dams from https://sigel.aneel.gov.br/Down/.

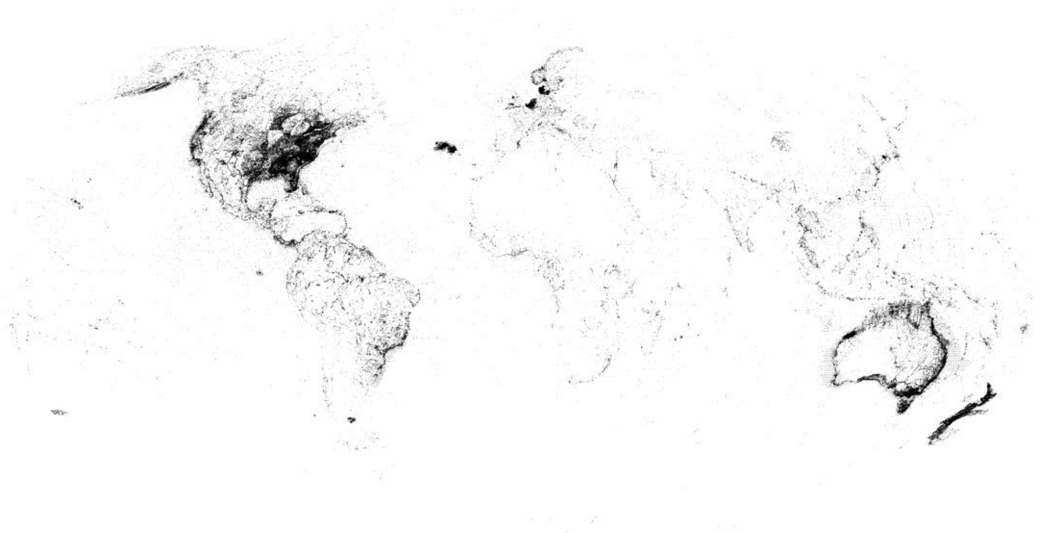

Figure S1. Spatial distribution of the occurrence records collected from the different datasets listed in Table S1. The point occurrence records were then converted to species-specific geographical ranges to complement the IUCN geographical ranges data with species not listed by IUCN.

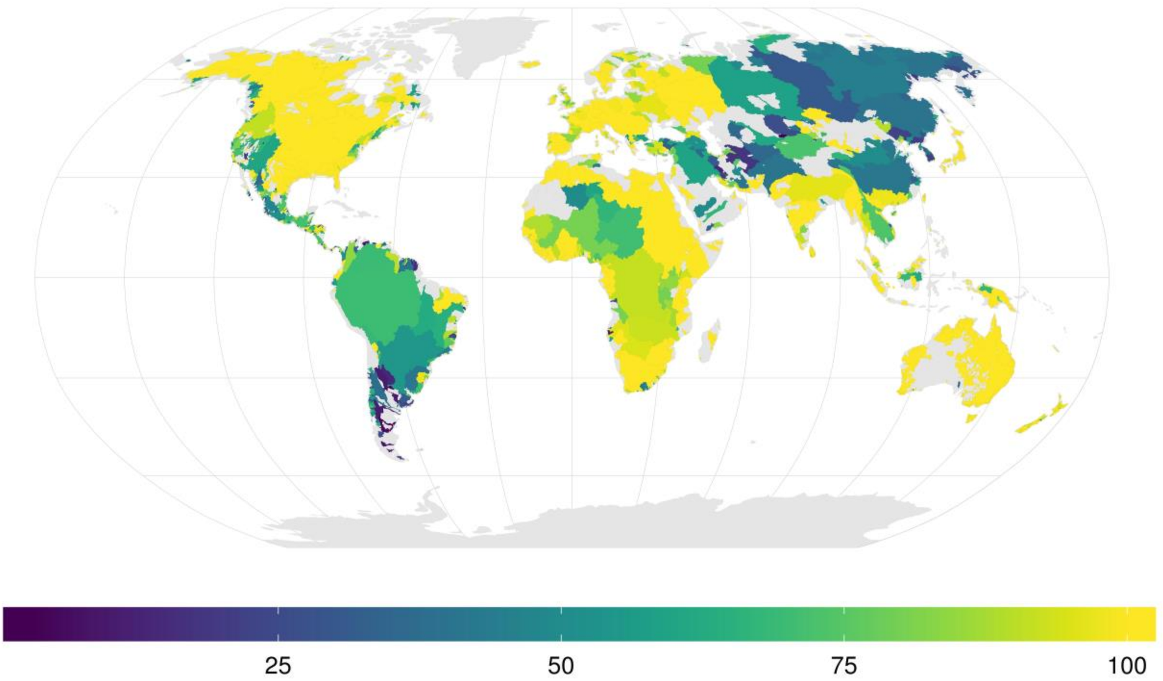

Figure S2. Ratio (%) between number of species of the dataset used in this study and the actual number of species according to Tedesco et al., (1) for the world's main hydrologic basins (i.e., with an outlet to the sea/internal sink). Main hydrologic basins with an equal or higher number of species than reported by (1) are set to 100% for representation purposes. Gray areas are basins not covered by the Tedesco et al., (1) dataset.

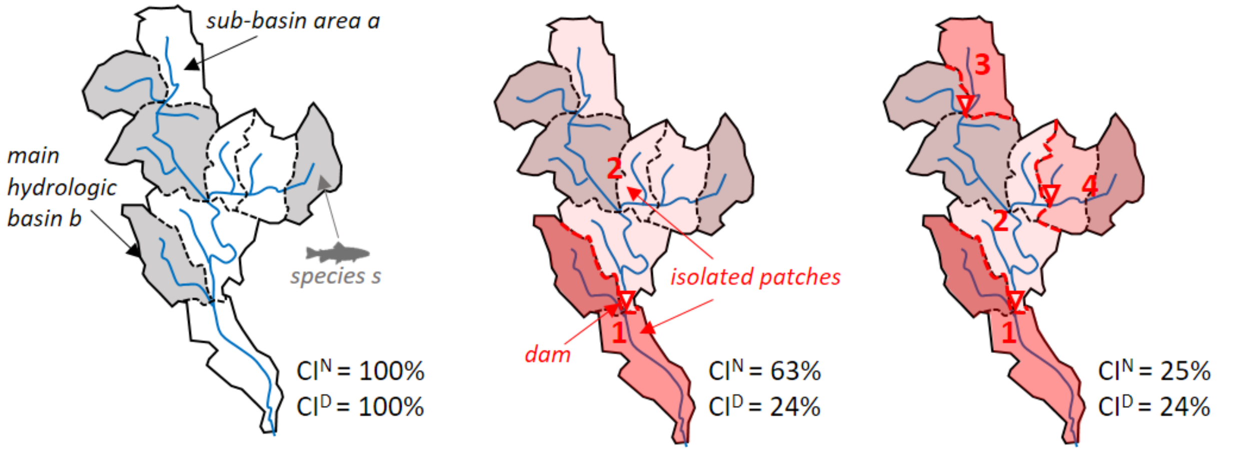

Figure S3. CI calculation for a hypothetical species *s* (occupying the gray areas) in a fictitious basin *b* (external solid line) partitioned in HB sub-basins (internal boundary dashed lines). The addition of dams fragments the basin in isolated patches (red hues with numbers). For each configuration, the CI is given for species *s* being either diadromous or non-diadromous. Note that the CI would not change for a diadromous species between the center and the right panel, even though the right panel contains more dams, as the connectivity for diadromous species is controlled by the most downstream dam.

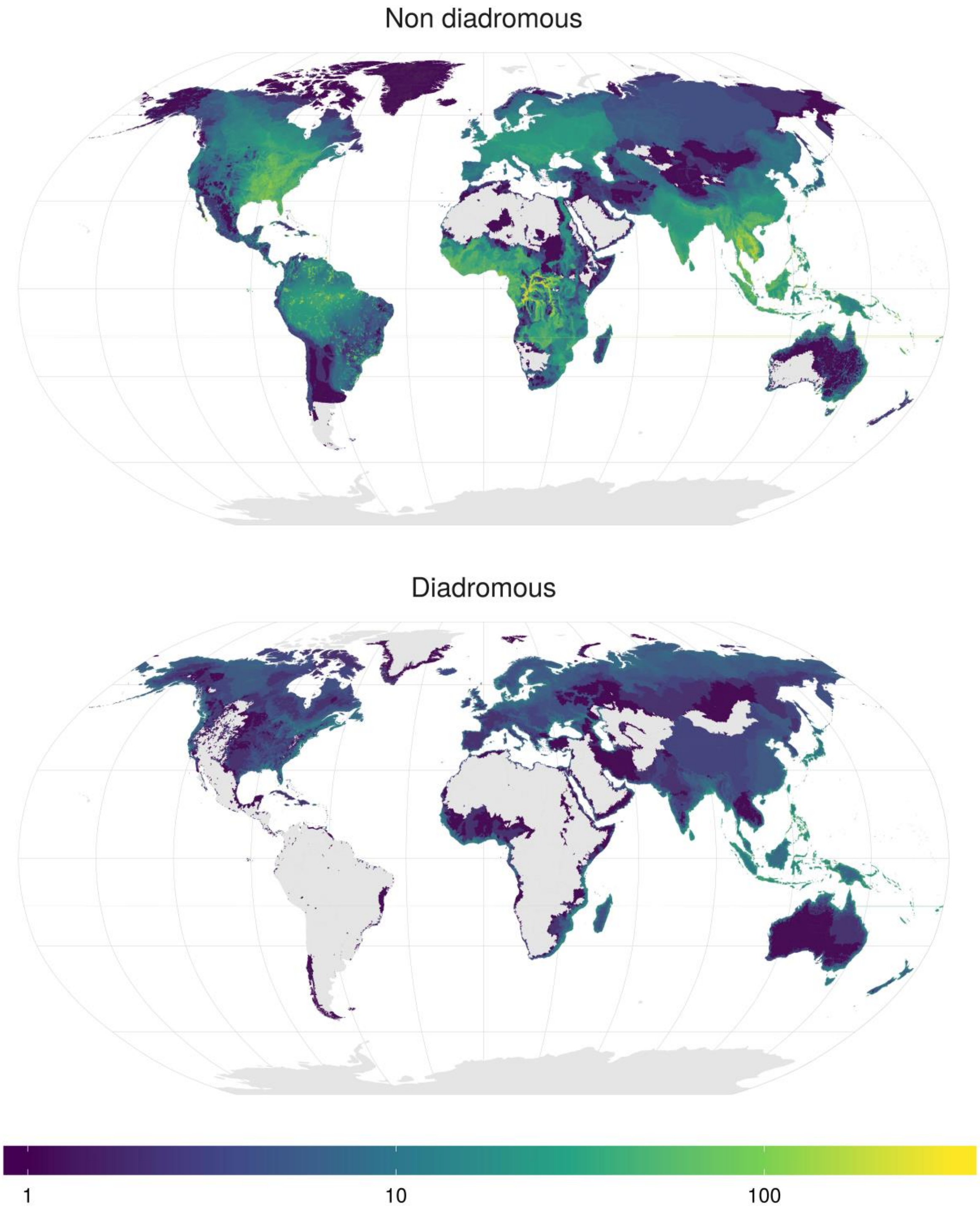

Figure S4. Occurrence data of 9,304 non-diadromous (top) and 490 diadromous (bottom) freshwater fish species included in this study, represented as the number of species in each of the ~1M sub-basins. Grey represents areas without species according to the IUCN database.

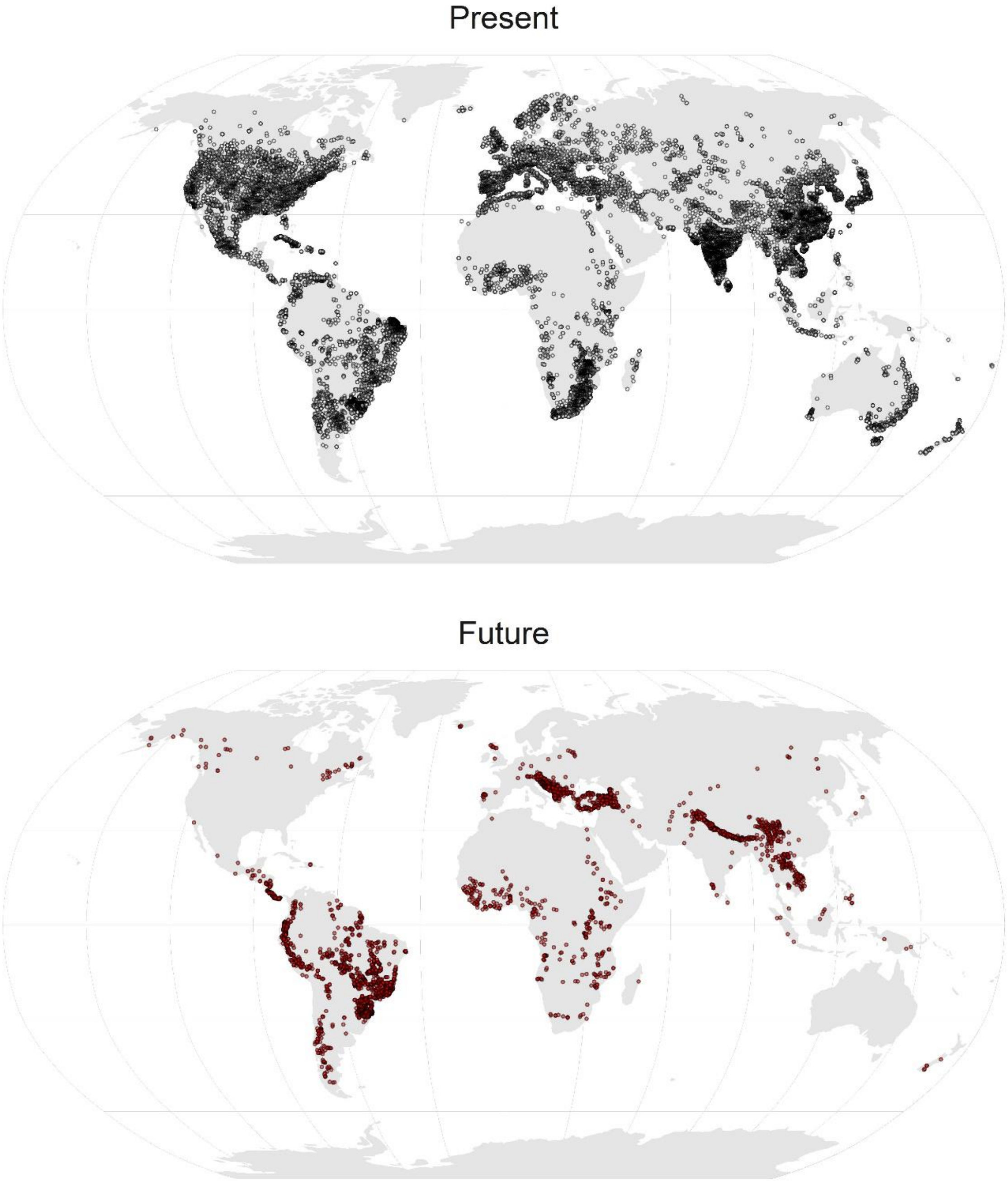

Figure S5. Location of dams included in this study for the present situation (39,912 dams; top) and for the future projection (3,681 dams; bottom).

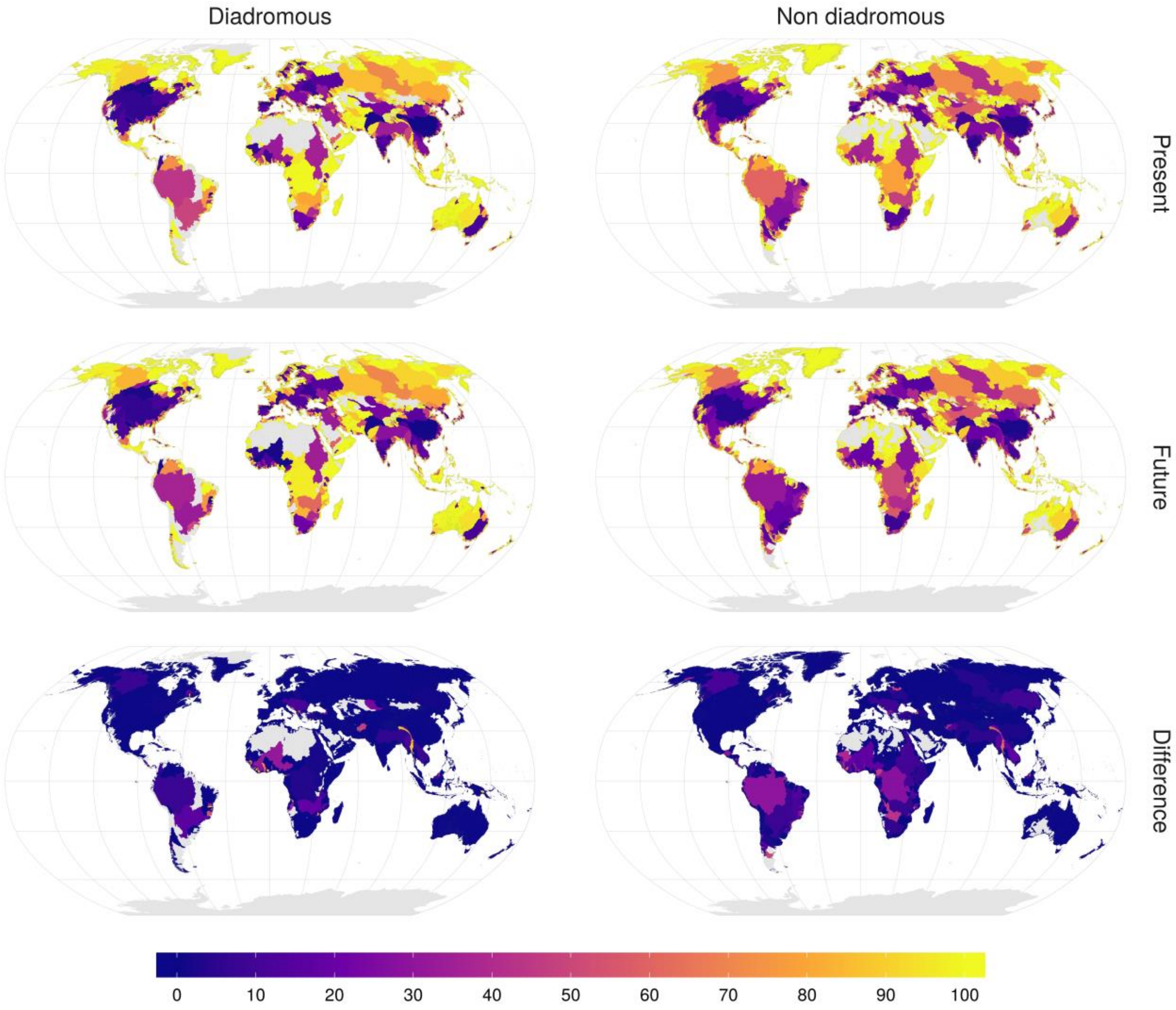

Figure S6. Connectivity index (%) for present and future scenarios aggregated for the world's main basins. The difference refers to the delta between present and future scenarios. For each of the ~58,000 basins, the mean of the CI value for the species occurring within that basin is reported. Grey represent areas without species according to the IUCN database.

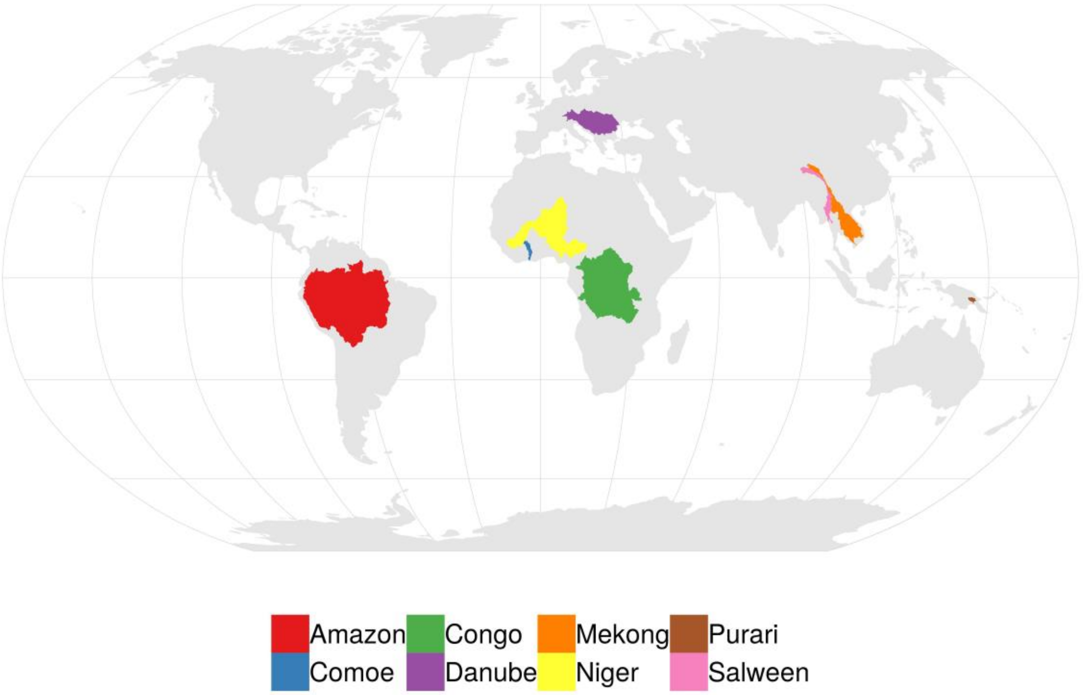

Figure S7. Location of example basins shown in Figures 3 and 4.

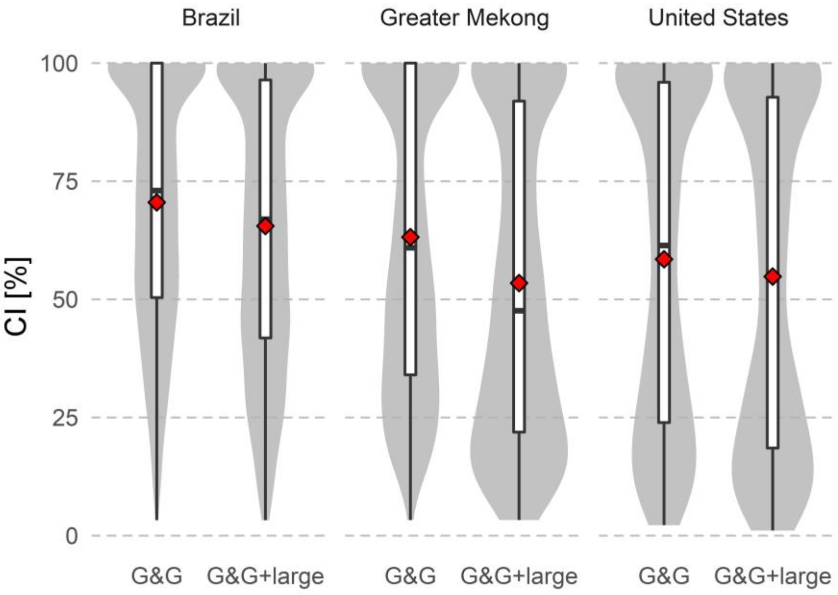

Figure S8. Representability of the global dataset of dams employed in this study (G&G) compared to national dams datasets (see Supplementary Methods). We used 498, 229 and 5,733 additional large dams with an height >15m for Brazil, the greater Mekon and the US, respectively. Boxes represent the interquartile range and the median, and whiskers the 95% interval. Gray areas around the boxes show the values distribution. The red diamonds represent the mean.

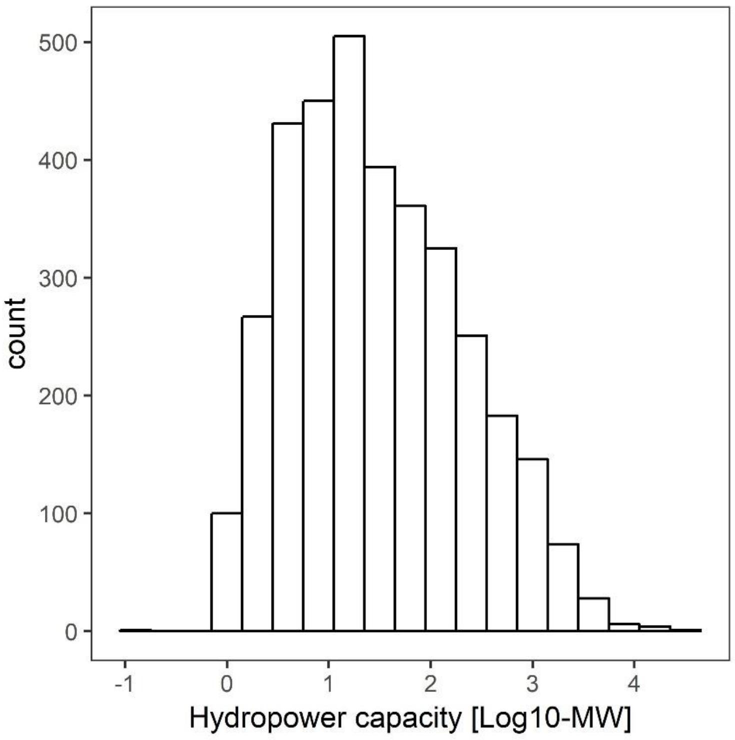

Figure S9. Hydropower capacity in log10-transformed MW based on the metadata available for future dams (8). Median = 23 MW, IQR = 109 MW, n = 3,527.

Table S1. Overview of source data used for the compilation of the global dataset of fish species occurrence records. For each source the number of species along with the total number of records is reported. All datasets are freely accessible and the code used to extract the records is available at https://github.com/vbarbarossa/occ2range4fish.

Source	Extent	No. species	No. records
**https://www.gbif.org**	Global	7,275	863,729
**http://www.fishnet2.net/**	Global	12,650	1,106,799
**http://splink.cria.org.br/**	Brazil	957	7,192
**https://portaldabiodiversidade.icmbio.gov.br/**	Brazil	2,490	42,939
**https://www.ala.org.au**	Australia	1,182	415,215

Table S2. Abbreviations used for the species order names of Figure 5i in the main text. “Other” groups together order names with less than 20 species available for our analysis.

ORDER NAME	ABBREVIATION USED
**Acipenseriformes**	Other
**Albuliformes**	Other
**Amiiformes**	Other
**Anguilliformes**	Angui.
**Ateleopodiformes**	Other
**Atheriniformes**	Ather.
**Aulopiformes**	Other
**Batrachoidiformes**	Other
**Beloniformes**	Belon.
**Beryciformes**	Other
**Carcharhiniformes**	Other
**Ceratodontiformes**	Other
**Characiformes**	Chara.
**Clupeiformes**	Clupe.
**Cypriniformes**	Cypri.
**Cyprinodontiformes**	Cypri.
**Elopiformes**	Other
**Esociformes**	Other
**Gadiformes**	Other
**Gasterosteiformes**	Other
**Gobiesociformes**	Other
**Gonorynchiformes**	Gonor.
**Gymnotiformes**	Gymno.
**Heterodontiformes**	Other
**Lepidosireniformes**	Other
**Lepisosteiformes**	Other
**Lophiiformes**	Other
**Mugiliformes**	Mugil.
**Myctophiformes**	Other
**Myliobatiformes**	Other
**Ophidiiformes**	Other
**Orectolobiformes**	Other
**Osmeriformes**	Osmer.
**Osteoglossiformes**	Osteo.
**Perciformes**	Perci.
**Percopsiformes**	Other
**Petromyzontiformes**	Petro.
**Pleuronectiformes**	Pleur.
**Polypteriformes**	Other
**Pristiformes**	Other

**Rajiformes**	Other
**Salmoniformes**	Salmo.
**Scorpaeniformes**	Scorp.
**Siluriformes**	Silur.
**Squaliformes**	Other
**Synbranchiformes**	Synbr.
**Syngnathiformes**	Syngn.
**Tetraodontiformes**	Tetra.
**Torpediniformes**	Other
**Zeiformes**	Other

References

1. P. A. Tedesco, *et al.*, Data Descriptor: A global database on freshwater fish species occurrence in drainage basins. *Sci. Data* **4**, 1–6 (2017).

2. R. Froese, D. Pauly, FishBase. *World Wide Web Electron. Publ.* (2018) (February 11, 2018).

3. B. Lehner, G. Grill, Global river hydrography and network routing: Baseline data and new approaches to study the world's large river systems. *Hydrol. Process.* **27** (2013).

4. IUCN, The IUCN Red List of Threatened Species. Version 2018-2 (2018) (November 13, 2018).

5. B. Lehner, K. Verdin, A. Jarvis, New global hydrography derived from spaceborne elevation data. *Eos (Washington. DC).* **89**, 93–94 (2008).

6. D. Cote, D. G. Kehler, C. Bourne, Y. F. Wiersma, A new measure of longitudinal connectivity for stream networks. *Landsc. Ecol.* **24**, 101–113 (2009).

7. J. T. Hack, F. A. Seaton, T. B. Nolan, Studies of Longitudinal Stream Profiles in Virginia and Maryland UNITED STATES DEPARTMENT OF THE INTERIOR (1957).

8. C. Zarfl, A. E. Lumsdon, J. Berlekamp, L. Tydecks, K. Tockner, A global boom in hydropower dam construction. *Aquat. Sci.* **77**, 161–170 (2015).
